# Late-Onset Schizophrenia (≥ 50 Years): A Retrospective Study of 30 Hospitalized Psychiatric Cases

**DOI:** 10.1192/j.eurpsy.2026.12204

**Published:** 2026-07-31

**Authors:** R. Rahoua, H. Zizi, M. Berghalout, I. Adali, F. Manoudi

**Affiliations:** Psychiatry, Ibn Nafis Hospital – University Hospital Center Mohammed VI, Marrakech, Morocco

## Abstract

**Introduction:**

Late-onset schizophrenia (≥ 50 years) is a clinical entity that remains relatively under-studied. It has drawn the attention of many researchers who have attempted to clarify its definition and characteristics. While most authors agree on defining it as a clinical presentation beginning after the age of 40, there is still disagreement regarding its specific clinical features and progression.(Yasuda M et al.Late-onset schizophrenia; 2013 Apr; 71(4):707-11)

**Objectives:**

To describe the clinical, therapeutic, and evolutionary characteristics of elderly patients hospitalized for late-onset schizophrenia.

**Methods:**

This is a descriptive retrospective study involving 30 patients aged 60 and over, hospitalized in the psychiatry department of Ibn Nafis Hospital – University Hospital Center Mohammed VI in Marrakech, between January 2024 and July 2025, with a diagnosis of schizophrenia whose first symptoms appeared after the age of 50. Clinical, sociodemographic, therapeutic, and outcome- related data were collected from medical records.

**Results:**

The average age of the patients was 62 years, with a female predominance (59%). The onset was insidious in 48.7% of cases. Persecutory delusions were the most frequently reported themes (74.3%), while auditory hallucinations were the most common delusional mechanism (81.2% of cases). Negative symptoms were observed in only 13% of patients. The majority of patients (86%) showed a good response to antipsychotic treatment, with a preference for atypical antipsychotics. Risperidone was prescribed in 48.3% of cases. Adverse effects were noted in 23.7% of cases.

**Conclusions:**

Late-onset schizophrenia is characterized by specific clinical features and often follows a more stable course than early-onset forms. Better recognition of this entity is essential to avoid diagnostic delays and to tailor therapeutic strategies to the aging population.(Wynn Owen PA et al. Late-onset schizophrenia: epidemiology, diagnosis, management and outcomes. Drugs Aging. 1999 Aug;15(2):81-9).

**Disclosure of Interest:**

None Declared

